# *Cicer arietinum* L. Sprouts’ Influence on Mineralization of Saos-2 and Migration of MCF-7 Cells

**DOI:** 10.3390/molecules25194490

**Published:** 2020-09-30

**Authors:** Małgorzata Zakłos-Szyda, Ilona Gałązka-Czarnecka, Joanna Grzelczyk, Grażyna Budryn

**Affiliations:** 1 Faculty of Biotechnology and Food Sciences, Institute of Molecular and Industrial Biotechnology, Lodz University of Technology, Stefanowskiego 4/10, 90-924 Lodz, Poland; 2 Faculty of Biotechnology and Food Sciences, Institute of Food Technology and Analysis, Lodz University of Technology, Stefanowskiego 4/10, 90-924 Lodz, Poland; ilona.czarnecka@p.lodz.pl (I.G.-C.); joanna.grzelczyk@dokt.p.lodz.pl (J.G.); grazyna.budryn@p.lodz.pl (G.B.)

**Keywords:** *Cicer arietinum* L., isoflavonoids, osteogenesis, proliferation, migration, apoptosis

## Abstract

In the present study, we investigated the biological activity of four extracts obtained from *Cicer arietinum* L. sprouts. The fermentation of the sprouts with *Lactobacillus casei* and their incubation with β-glucosidase elevated the concentrations of isoflavonoids, especially coumestrol, formononetin and biochanin A. To study the biological activity of *C. arietinum*, the human osteosarcoma Saos-2 and human breast cancer MCF-7 cell lines were used. The extracts obtained from fermented sprouts exhibited the strongest ability to decrease intracellular oxidative stress in both types of cells. They augmented mineralization and alkaline phosphatase activity in Saos-2 cells, as well as diminished the secretion of interleukin-6 and tumor necrosis factor α. Simultaneously, the extracts, at the same doses, inhibited the migration of MCF-7 cells. On the other hand, elevated concentrations of *C. arietinum* induced apoptosis in estrogen-dependent MCF-7 cells, while lower doses stimulated cell proliferation. These results are important for carefully considering the use of fermented *C. arietinum* sprouts as a dietary supplement component for the prevention of osteoporosis.

## 1. Introduction

**Figure 1:**
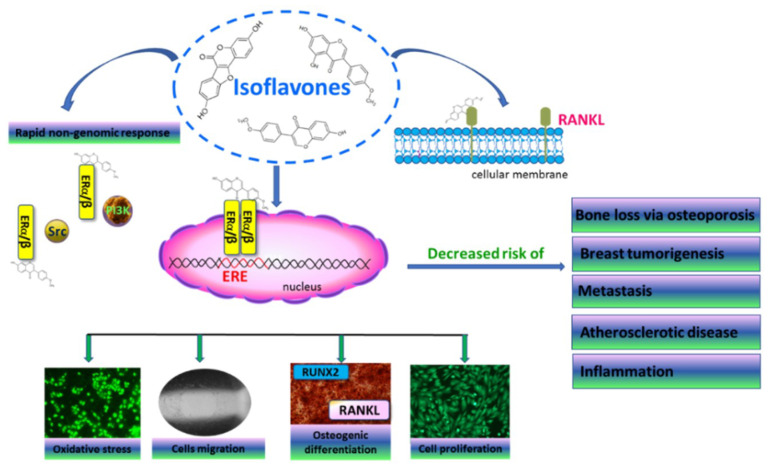
The main health-promoting activities of isoflavones with regard to the regulation of bone tissue mineralization and breast tumorigenesis. ERα/β—estrogen receptor α/β; ERE—estrogen response element; PI3K—PI3 kinase; RANKL—receptor activator of nuclear factor-κB ligand; RUNX2—Runt-related transcription factor 2; Src—Src kinase.

There is growing evidence showing the beneficial properties of plant-originated dietary components for disease prevention [1]. In addition to several nutrients, the most relevant phytocompounds affecting human health are phenolic compounds. These plant secondary metabolites possess strong antioxidant properties and have been proven to modulate cell-signal transduction at the molecular level during in vitro and in vivo experiments [1,2,3,4,5,6]. Their biological activity is strongly associated with their chemical structure, amount and bioavailability, and epidemiological studies have correlated the uptake of phenolics, especially isoflavones, with a lower incidence of osteoporosis and breast cancer [7,8,9]. Since the chemical structure of isoflavones resembles estrogen, these compounds are known as phytoestrogens. Estrogens significantly participate in bone-tissue mineralization, lipid metabolism and cardioprotection, and their deficiency, especially after menopause, significantly increases the risk of atherosclerotic disease and osteoporosis [9]. They are natural ligands of nuclear estrogen receptors (ER), which act as transcription factors by binding to the estrogen response element (ERE), and regulate the expression of other genes related with cell proliferation, migration and differentiation (Figure 1) [10]. Despite this classical action, estrogen receptors can activate a rapid non-genomic response through interaction with other signaling proteins and enhancing cell proliferation or migration [11]. Two isoforms of estrogen receptors, ERα and ERβ, are known and often have opposite transcriptional effects: ERα activation leads to enhanced cell proliferation, whereas ERβ activation leads to its reduction [12]. Bone tissue homeostasis is regulated by osteoclasts, which reduce the mineralized extracellular matrix, and osteoblasts, which produce organic bone mass [13].

Estrogen-receptor activation in bone-tissue cells regulates the expression of Runt-related transcription factor 2 (RUNX2), a master regulator of osteogenic differentiation [14]; estrogen deficiency reduces osteogenesis and promotes bone resorption and osteoporosis [15]. Moreover, the suppression of receptor activator of nuclear factor-κB ligand (RANKL) expressed by osteoblasts, as well as the secretion of bone-resorbing cytokines, prevents osteoclast activation. Thus, hormone-replacement therapy inhibits the loss of bone mass, but also elevates the risk of estrogen-induced tumorigenesis, especially breast and endometrial cancer [8]. It is known that estrogen receptor α has a higher binding affinity for estrogens, whereas ERβ can bind other ligands with higher affinity than the α isoform [16]. Therefore, ER ligands able to maintain bone structure without proliferative effects on breast cancer cells may be a potent tool for the prevention of bone-mass loss and the development of breast tumorigenesis. Studies performed with isoflavones have shown that they have a higher binding affinity for ERβ than ERα [16], which indicates that they are able to bind to estrogen receptors and attenuate bone loss without inducing tumorigenesis, due to their weak estrogenic potential [7]. It is therefore considered that a diet rich in phytoestrogens can reduce the symptoms of estrogen deficiency. Among the richest plant sources of isoflavones are legumes such as soybeans, which contain genistein [17]. Previous study revealed that the sprouts of *Cicer arietinum* L., known as chickpeas, contained high amounts of formononetin and biochanin A, different types of isoflavones [17]. According to the literature, *C. arietinum*’s isoflavone content varies from 153 to 340 mg/100 g [18]. This plant is mainly grown in Asia and is present in the human diet as cooked grains or grain flour [19]. The chickpea seeds are known as a traditional Uighur herb and medicine in China, which has been used to prevent hypertension, hyperlipidemia, diabetes, itchy skin, flatulence, low libido, tumor formation and osteoporosis [13]. While the antioxidative and inflammation-regulating potential of chickpeas has been reported [18], there has been no study about the influence of *C. arietinum*-sprout isoflavonoids on bone-cell mineralization.

The aim of the present study was to evaluate the biological activity of *C. arietinum*’s sprouts after their fermentation with *Lactobacillus casei* and their incubation with β-glucosidase. To assess *C. arietinum*’s influence on the mineralization process, the human osteosarcoma Saos-2 cell line was used. Since oxidative stress and chronic inflammation stimulate bone demineralization, the extracts’ impact on intracellular oxidative-stress generation, as well as interleukin-6 (IL-6) and tumor necrosis factor α (TNF-α) secretion, in Saos-2 cells was assessed. Taking into account that bone tissue is one of the most common metastatic sites in breast cancer, the influence of *C. arietinum* on human breast-cancer MCF-7 cell migration and cell-death induction was also investigated.

## 2. Results

### 2.1. Isoflavone Profile According to LC-ESI-MS Analysis

**Figure 2:**
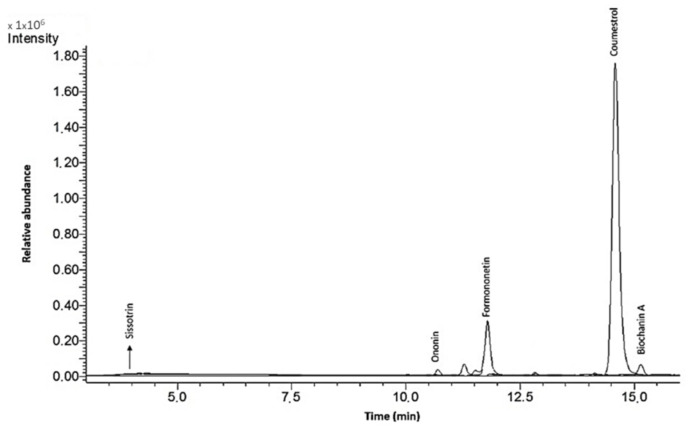
An example of an LC/MS chromatogram of the main isoflavones contained in the chickpea (*Cicer arietinum* L.) sprouts extract ChUF. The m/z values recorded on the graph were as follows: 269 for coumestrol, 270 for formononetin, 285 for biochanin A, 431 for ononin and 447 for sissotrin.

*C arietinum* sprouts are a very rich source of isoflavones and could be used to supply dietary phytoestrogens together with soybeans [18]. The time and intensity of UVB-light exposure optimal for sprout growth and isoflavone content were determined in previous research [16,20]. The lactic acid fermentation of sprouts with *Lactobacillus casei* 0979 strain and β-glucosidase treatment was performed to increase the isoflavone content and increase the aglycone forms [17]. For the biological studies, four different extracts obtained from *C. arietinum* L. sprouts treated with lactic fermentation or incubated with β-glucosidase were investigated: chickpea extract (ChU), extract incubated with β-glucosidase (ChUH), extract obtained from sprouts after fermentation (ChUF) and extract obtained from fermented sprouts after incubation with β-glucosidase (ChUFH). The results of the qualitative and quantitative analysis of the isoflavonoids in *C. arietinum* extracts by LC-ESI-MS are presented in Table 1; an example of an LC/MS chromatogram is shown in Figure 2.

**Figure 3:**
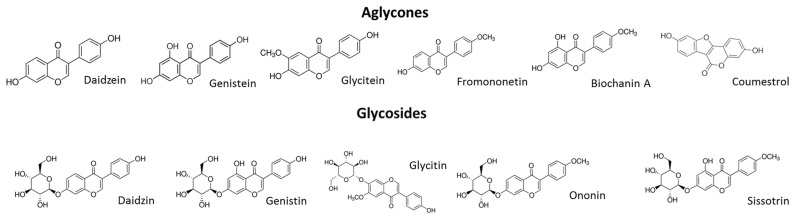
Chemical structures of main isoflavonoids present in *C. arietinum* sprouts.

The total isoflavonoid content varied from 490.3 to 2095.0 mg/100 g of dry mass of sprouts. In the dry mass of the extract, the total isoflavonoid content varied from 1229.2 to 3623.8 mg/100 g. The main isoflavones are the glycosides ononin and sissotrin (Figure 3). The main aglycones identified in *C. arietinum* were formononetin and biochanin A, whereas sprout fermentation led to elevated aglycone production, with coumestrol, formononetin and biochanin A as the main identified compounds. In both cases, fermentation significantly increased the isoflavone content. Interestingly, an almost 40-fold increase in the coumestrol concentration in the ChUF and ChUFH preparations was observed, a phenomenon previously confirmed in fermented soybean sprouts [21]. Hydrolysis decreased the glycoside content in the ChUH and ChUFH samples, leading to an increase in aglycone content.

### 2.2. C. arietinum L.’s Effects on Cell Metabolic Activity

**Figure 4:**
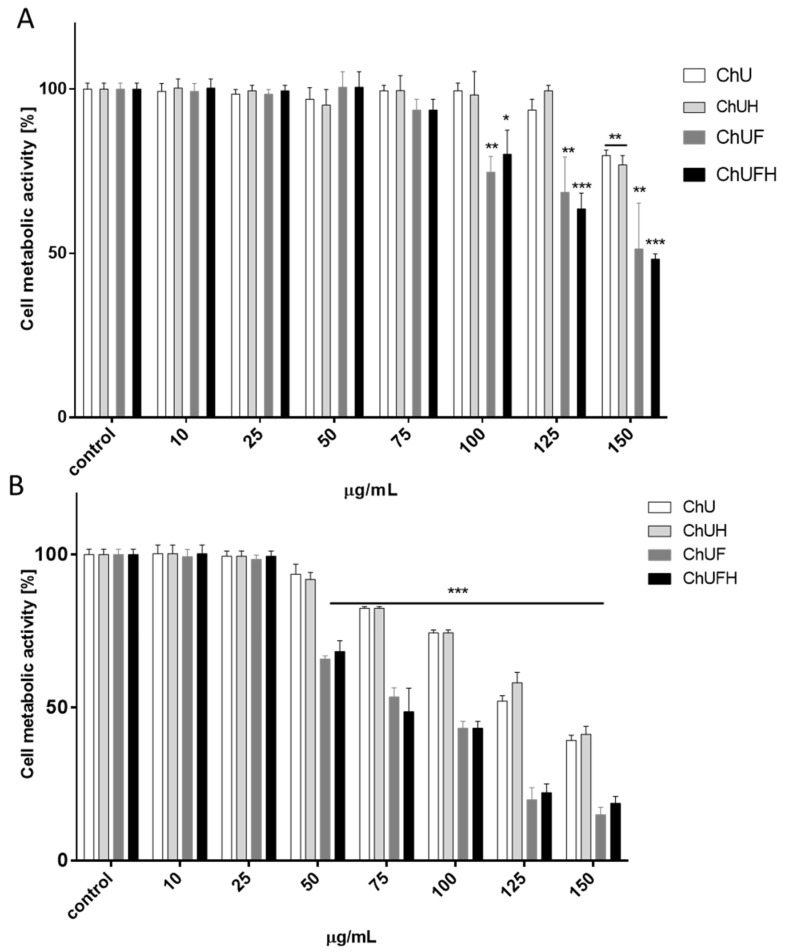
The influence of *C. arietinum* extracts on Saos-2 (**A**) and MCF-7 (**B**) cell metabolic activity as determined by the Presto Blue assay after 48 h of exposure to ChU, ChUH, ChUF and ChUFH. Control cells were not exposed to any compound except vehicle; values are means ± standard deviations, *n* ≥ 9; * *p* ≤ 0.05, ** *p* ≤ 0.01, *** *p* ≤ 0.001.

To assess the cytotoxic potential of *C. arietinum* preparations, Saos-2 and MCF-7 cells were incubated with concentrations of 10–150 µg/mL for 48 h. As presented in Figure 4, cell metabolic activity decreased with increasing extract concentrations.

The MCF-7 cells were more sensitive to the studied preparations than Saos-2 cells. ChUF and ChUFH showed the greatest cytotoxic potential. The treatment of MCF-7 cells with 50 µg/mL of both preparations decreased metabolic activity by almost 35%. In the osteosarcoma cells, a decrease in metabolic activity was detected after treatment with 100 µg/mL of both samples. The unfermented ChU and ChUH extracts had less influence on the metabolic activity of both of the studied cell lines: the highest concentration decreased Saos-2′s metabolic activity to 80% and MCF-7′s to 40%. In summary, the *C. arietinum* extracts obtained from unfermented sprouts had lower cytoxicity towards the studied cellular models.

The IC_50_ values of the fermented *C. arietinum* preparations were approximately 75 and 150 µg/mL for MCF-7 and Saos-2 cells, respectively. Because we intended to simultaneously assess the effect of *C. arietinum* on both types of cells, we chose the highest non-cytotoxic concentration (IC_0_), 25 µg/mL, for further study.

**Figure 5:**
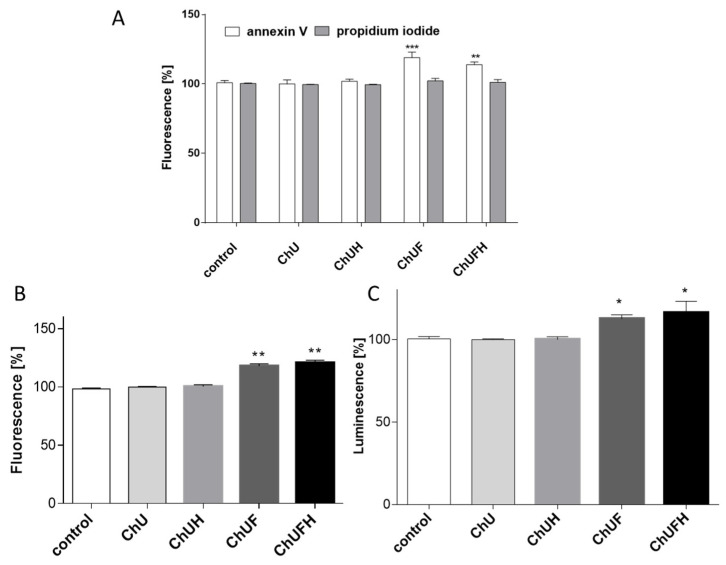
The influence of *C. arietinum* preparations at 40 µg/mL on phosphatidylserine externalization on the outer membrane leaflet of apoptotic MCF-7 cells and membrane permeabilization detected with an annexin-V-FITC assay kit and propidium-iodide staining (**A**); caspase-3/7 activation (**B**); caspase-9 activation (**C**). Control cells were not exposed to any compound except the vehicle; values are means ± standard deviations, *n* ≥ 3; statistical significance was calculated versus control cells (exposed to vehicle), * *p* ≤ 0.05, ** *p* ≤ 0.01, *** *p* ≤ 0.001.

Due to the high cytotoxicity of the fermented preparations towards MCF-7 cells, we studied their influence at 40 µg/mL, which is higher than the IC_0_ but below the IC_50_ value. The experimental data presented in Figure 5A confirmed that the fermented samples increased the externalization of annexin V on the cell membrane by 15–20% without damaging membrane integrity. This was accompanied by a 20% activation of caspase-3/7 (Figure 5B), and an almost 10% elevation of the luminescence generated by active caspase-9 (Figure 5C).

### 2.3. C. arietinum L.’s Influence on Saos-2 Mineralization

**Figure 6:**
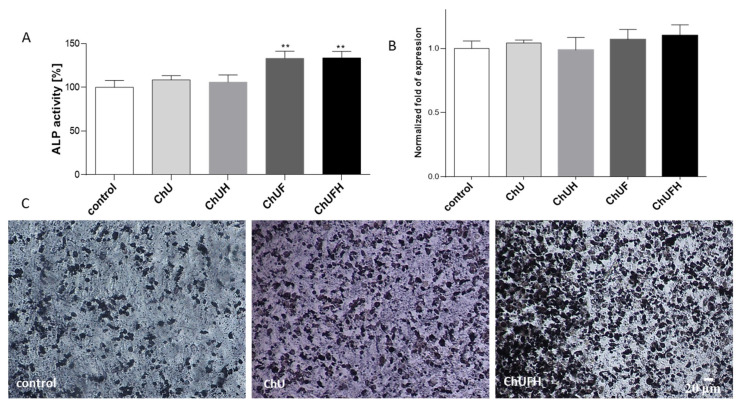
The influence of the C. arietinum extracts at IC_0_ doses on ALP activity (**A**) and ALP mRNA (**B**) in Saos-2 cells after 7 days of exposure; control cells were not exposed to any compound except vehicle; values are means ± SEM, *n* ≥ 4. Microscopic observation of cells with active ALP (in the presence of BCIP/NBT) after treatment with ChU and ChUFH (**C**); randomly chosen fields were photographed at ×200 with a contrast-phase microscope. Statistical significance was calculated versus control cells (exposed to vehicle) with ** *p* ≥ 0.01.

The osteoblasts’ mineralization in Saos-2 cells is manifested by an elevation of alkaline phosphatase (ALP) activity [22]. Studies demonstrated that ChUF and ChUFH extracts augmented ALP activity by nearly 30% (Figure 6A), whereas no relevant effect on the *ALP* mRNA expression level was observed (Figure 6B). The extracts obtained from unfermented sprouts had less effect than the fermented ones, and increased ALP activity by approximately 5–7%. Microscopic observations of Saos-2 cells after incubation with an ALP substrate (BCIP/NBT) confirmed that the fermented samples were the strongest activators of cellular ALP (Figure 6C).

**Figure 7:**
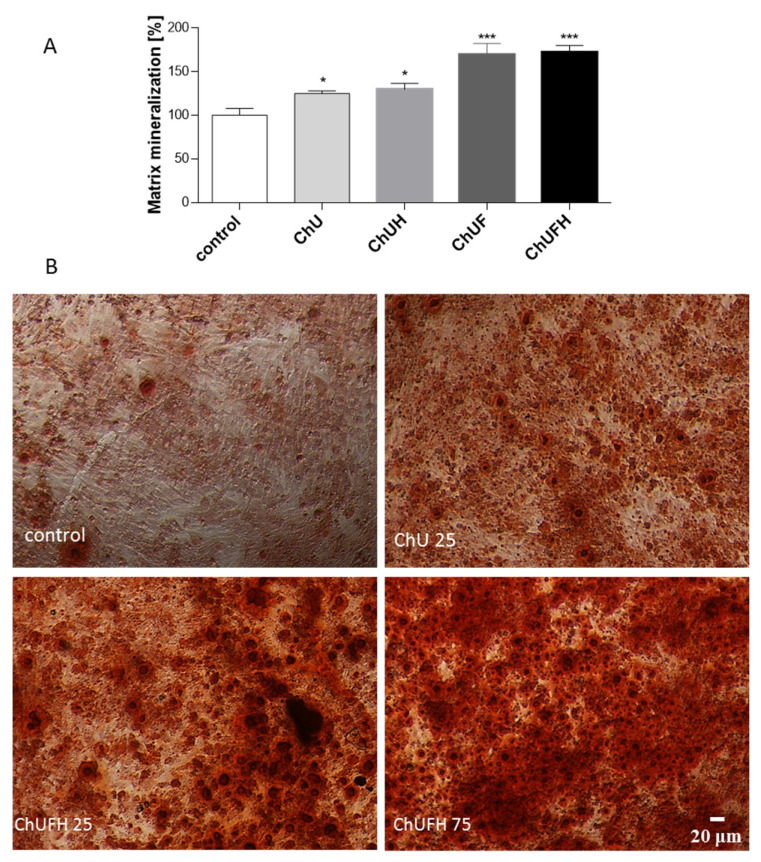
The influence of *C. arietinum* extracts at IC_0_ doses on Saos-2 cell-matrix mineralization after 7 days of exposure (**A**), cells stained with Alizarin Red S after treatment with ChU and ChUFH at 25 µg/mL (IC_0_ dose) and ChUFH at 75 µg/mL (**B**); randomly chosen fields were photographed at ×200 with a contrast-phase microscope. Control cells were not exposed to any compound except vehicle; values are means ± SEM, *n* ≥ 4; statistical significance was calculated versus control cells (exposed to vehicle), * *p* ≥ 0.05, *** *p* ≥ 0.001.

Figure 7A illustrates that matrix mineralization was increased in cells treated with all the *C. arietinum* extracts. The ChU and ChUH samples intensified the matrix mineralization process by 25%, whereas the preparations from fermented sprouts elevated that process by 75% in comparison with that in the control cells. These results were in agreement with the microscopic observations (Figure 7B). The preparations augmented the number of red-stained round-shaped granules, indicating bone-nodule formation and thus the stimulation of Saos-2 osteogenic differentiation.

**Figure 8:**
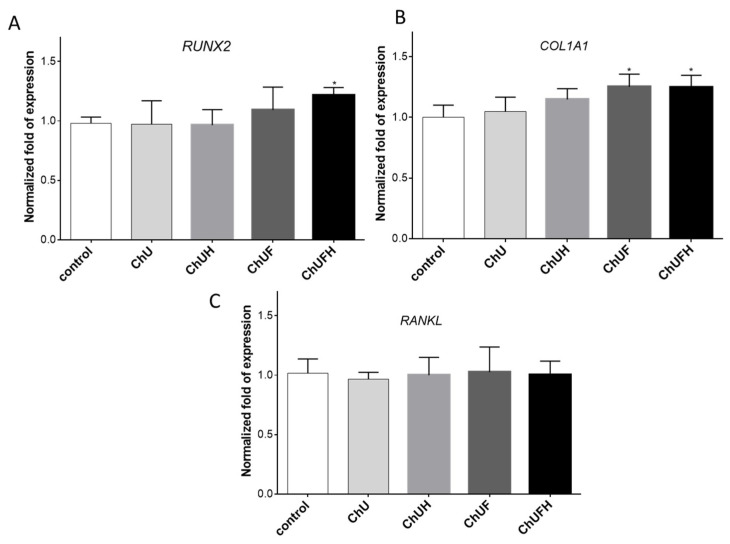
The influence of *C. arietinum* extracts at IC_0_ doses on the mRNA expression of *RUNX2* (**A**), *COL1A1* (**B**) and *RANKL* (**C**) genes in Saos-2 cells after 7 days of exposure. Control cells were not exposed to any compound except vehicle; values are means ± SEM, *n* = 4; statistical significance was calculated versus control cells (exposed to vehicle), * *p* ≥ 0.05.

As presented in Figure 8A, the elevation of Runt-related transcription factor 2 (*RUNX2)* was observed in cells treated with fermented extracts, whereas only ChUFH extract elevated its mRNA level, by 15%, with statistical significance. Similarly, the extracts obtained from fermented sprouts upregulated the mRNA level of type 1 collagen (*COL1A)* by approximately 15% in comparison to that in the control cells (Figure 8B). The receptor activator of nuclear factor kappa-Β ligand (RANKL) protein present on the surface of osteoblasts, after binding with the RANKL receptor present on osteoclasts, activates osteoclastogenesis. As can be seen in Figure 8C, *RANKL* gene expression was not affected by preparations at the transcriptional level.

**Figure 9:**
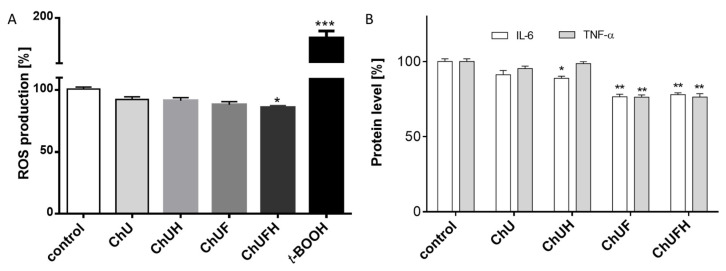
The influence of *C. arietinum* at IC_0_ dose on intracellular ROS generation in Saos-2 cells; as a positive control for ROS generation, 500 µM *t*-BOOH was used (**A**). The levels of TNF-α and IL-6 secretion in Saos-2 cells (**B**). Control cells were not exposed to any compound except vehicle; sample-influence values are means ± SEM, *n* ≥ 4; statistical significance was calculated versus control cells; * *p* ≤ 0.05, ** *p* ≤ 0.01, *** *p* ≤ 0.001.

The pre-incubation of Saos-2 cells with samples at IC_0_ concentrations decreased intracellular reactive oxygen species (ROS) by 5–15% compared to those in the cells treated with the vehicle only (Figure 9A). The fermented preparations were also revealed to possess greater radical-scavenging and antioxidant potential than the unfermented samples. As demonstrated in Figure 9B, all the samples downregulated the secretion of interleukin-6 by 5–20%. TNF-α secretion was diminished by almost 25% in the cells exposed to the ChUF and ChUFH extracts.

### 2.4. C. arietinum’s Effect on MCF-7 Cell Migration

**Figure 10:**
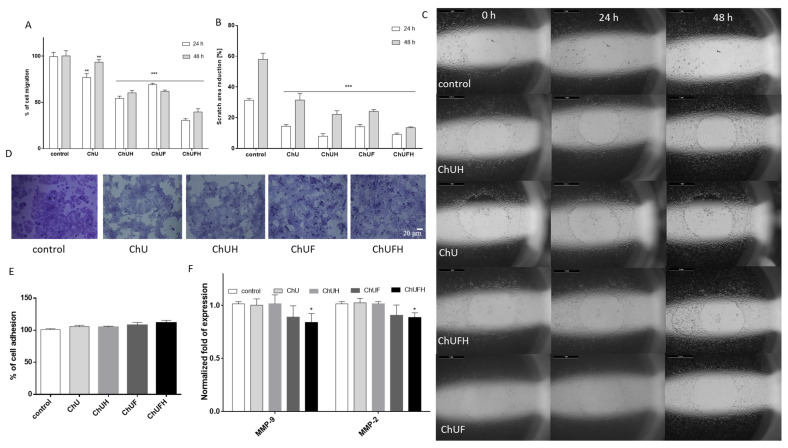
The influence of 48 h of exposure to the *C. arietinum* extracts at IC_0_ doses on the migration (**A**) and scratch-area reduction (**B**) of MCF-7 cells. For MCF-7 cells incubated with extracts at IC_0_ doses, the rates of migration into the free detection zones were photographed (×8) (**C**). Cell adhesion to the substrate randomly photographed (×200) (**D**) and measured after staining with crystal violet (**E**). The samples influenced the *MMP-9/2* mRNA expression level (**F**). Control cells were not exposed to any compound except the vehicle; values are means ± SD, *n* ≥ 4; statistical significance was calculated versus control cells; * *p* ≤ 0.05, ** *p* ≤ 0.01, *** *p* ≤ 0.001.

Bone tissue is one of the most common metastatic sites for breast cancer [20]; the effect of *C. arietinum* isoflavones on the detachment and migration of breast-cancer MCF-7 cells was assessed. As can be seen in Figure 10A–C, all the samples decreased the migration ratio of MCF-7 cells, as well as the scratch-area reduction: ChU had the least effect, whereas the highest activity was observed for ChUFH. That inhibition of migration was more effective after the first 24 h of treatment, where the migration percentage of the cells treated with ChUFH did not exceed 30% compared to the control. Nevertheless, the inhibitory effect was sustained after 48 h of cell incubation with the extracts.

Cell staining with crystal violet confirmed that the detachment of the MCF-7 cells was not involved in the reduction of the migration rate. Not only was the amount of dye comparable for all the experiments, but the morphology of the cells was not changed after treatment (Figure 10D,E). These observations allowed the hypothesis that the extracts’ constituents may influence the expression and activity of enzymes remodeling the extracellular matrix, such as matrix metalloproteases (MMPs). Among the different types of MMPs, the main proteinases involved in the degradation of the extracellular matrix’s collagen components are gelatinases known as MMP-2 and MMP-9 [23]. As presented in Figure 10F, only the fermented extracts downregulated the expression of *MMP-2* and *MMP-9* at the mRNA level, by 15–20%.

**Figure 11:**
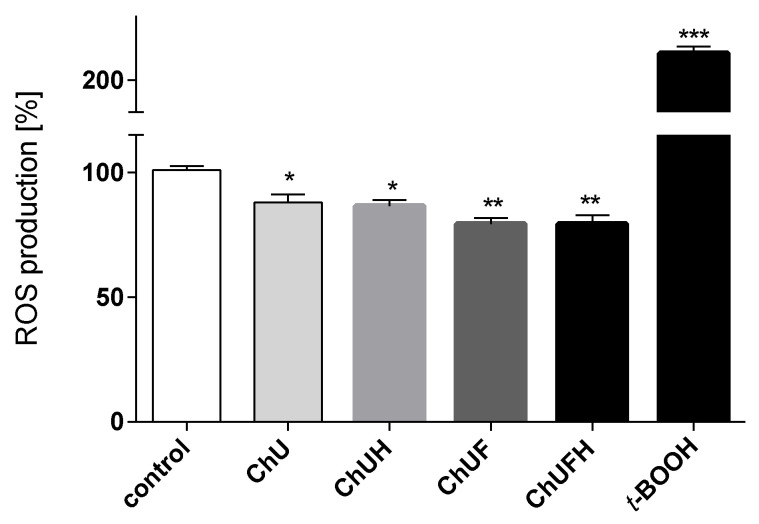
The effect of *C. arietinum* extracts at IC_0_ doses on intracellular ROS generation in MCF-7 cells; control cells were not exposed to any compound except vehicle; as a positive control for ROS generation, 500 µM *t*-BOOH was used; values are means ± standard deviations, *n* ≥ 4; statistical significance was calculated versus control cells, * *p* ≤ 0.05, ** *p* ≤ 0.01, *** *p* ≤ 0.001.

The increase in cell growth and proliferation may lead to hypoxia, which, in turn, elevates the generation of reactive oxygen species and metastasis [20]. As demonstrated in Figure 11, the pre-incubation of the MCF-7 cells with the extracts at IC_0_ doses diminished the intracellular ROS levels by 10–15% compared to those in the control cells. The ChUF and ChUFH extracts were stronger oxidative stress reducers than the unfermented samples.

**Figure 12:**
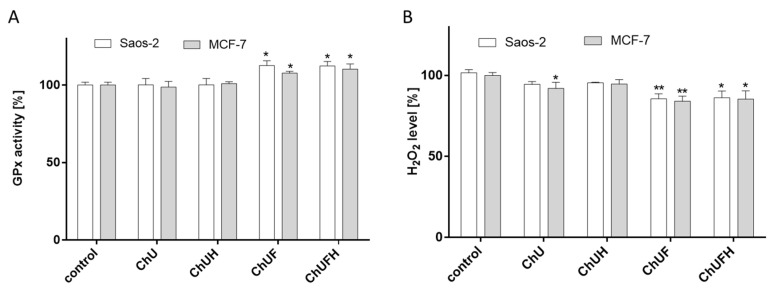
The effect of *C. arietinum* extracts at IC_0_ doses on GPx activity (**A**) and hydrogen peroxide level (**B**) in MCF-7 and Saos-2 cells; control cells were not exposed to any compound except vehicle; values are means ± standard deviations, *n* ≥ 4; statistical significance was calculated versus control cells, * *p* ≤ 0.05, ** *p* ≤ 0.01.

The important cellular intermediators promoting migration are ROS, especially H_2_O_2_. The *C. arietinum*’s effects on the activity of intracellular glutathione peroxidase (GPx), a protein that catalyzes the removal of free hydrogen peroxide and lipid hydroperoxides [24], was subsequently assessed. As presented in Figure 12A, incubation with the fermented extracts elevated GPx activity in MCF-7 cells by 10% in comparison to the control, accompanied by an almost 10% decrease in the hydrogen peroxide level (Figure 12B). As previously determined, both fermented samples had the strongest cytoprotective abilities. Comparable results showing cytoprotective properties were obtained for osteoblastic cells.

**Figure 13:**
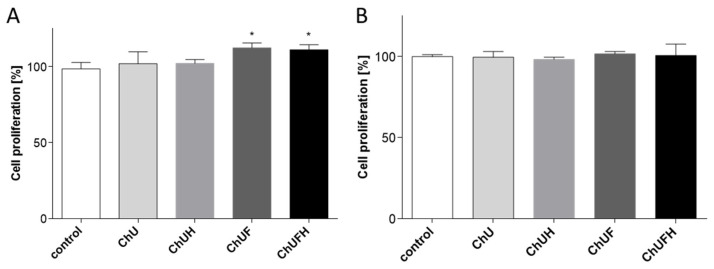
The effects of *C. arietinum* extracts at IC_0_ doses on MCF-7 (**A**) and Saos-2 (**B**) cell proliferation, determined with CyQuant reagent after 48 h incubation; control cells were not exposed to any compound except vehicle; values are means ± SEM, *n* ≥ 12; statistical significance was calculated versus control cells, * *p* ≤ 0.05.

MCF-7 cells are known to be estrogen-dependent [25]. Due to the structural similarity of isoflavones to estrogen, next, the effects of the *C. arietinum* extracts, at IC_0_ concentrations, on the proliferation of MCF-7 cells were evaluated. As shown in Figure 13A, the unfermented extracts did not induce MCF-7 cell proliferation with statistical significance, whereas the fermented samples elevated the cell division ratio by circa 10%. These results allowed the speculation that the extracts obtained from fermented sprouts had weak estrogenic activity. Simultaneously, a control experiment performed with Saos-2 revealed that the *C. arietinum* extracts at IC_0_ doses did not influence the proliferation rate of osteosarcoma cells (Figure 13B).

**Figure 14:**
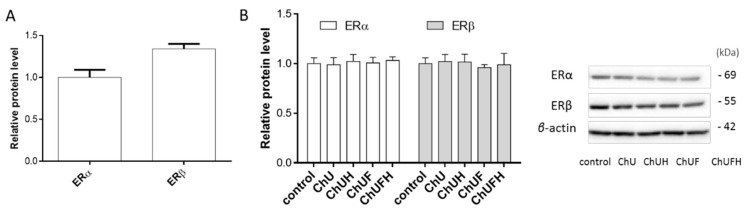
Analysis of the relative ERα and ERβ expression in MCF-7 cells (samples were calibrated by ERα) (**A**). The effects of *C. arietinum* extracts at IC_0_ doses on the expression of estrogen-receptor proteins in MCF-7 cells, normalized using *β*-actin as a reference protein (**B**). Bands of a representative Western blot experiment. Control cells were not exposed to any compound except vehicle; values are means ± SD, *n* = 3.

It was confirmed that the MCF-7 cells expressed both isoforms of estrogen receptors (Figure 14A) [25,26]. In the studied cells, the expression of the ERβ-receptor protein was almost 25% higher than that of the ERα isoform. The *C. arietinum* extracts did not influence the expression of either estrogen-receptor isoform after the incubation of MCF-7 cells for 48 h with extracts at IC_0_ doses (Figure 14B).

## 3. Discussion

In the present study, the biological activity of four extracts obtained from *Cicer arietinum* L. sprouts, which were fermented with the health-promoting *Lactobacillus casei*, or treated with β-glucosidase, was investigated. It must be emphasized that the isoflavonoid content was determined not only in preparations obtained from sprouts, but also in sprouts that can be eaten directly. The extractions from sprouts were performed to obtain data about the isoflavonoid concentration, as well as to achieve preparations that could be added to the cells for in vitro studies. A comparison of obtained data shows that the isoflavonoid content in sprouts is strongly elevated during fermentation. Due to the increase in protein and carbohydrate extraction efficiency, the advantage of fermentation for the extracts is lower than that for the sprouts. Still, the main isoflavonoids identified in the *C. arietinum* sprouts were formononetin, onionin and biochanin A.

In this work, human osteosarcoma Saos-2 cells were used, which resemble osteoblastic cells, expressing active alkaline phosphatase (ALP) and forming the calcified matrix [27]. The osteoblast differentiation process is manifested by an increase in matrix mineralization and bone-nodule formation. This process can already be observed in cells after 7 days of incubation in differentiation medium-containing osteogenic inducers. The intention of the study was to evaluate phytocompounds as osteogenesis activators without any toxic effects on cellular viability after the cell-differentiation period.

The direct assessment of all samples’ influence on cell metabolic activity showed that the almost 2.5-fold increase in the total isoflavonoids in the fermented sprout samples was followed by a 5-fold reduction of the active dose. Still, a comparison of the IC_0_ and IC_50_ values did not reveal a tenfold difference between the obtained concentrations, which may suggest potential toxic effects induced by the fermented samples. The evaluation of the biologically effective isoflavonoid concentration with data obtained for soy compounds (IC_0_ ≈ 12.5–50 µg/mL) reveals *C. arietinum* fermented sprout extracts as comparable modulators of cell metabolic activity [28].

The RUNX2 transcription factor is known as the key osteoblast differentiation regulator involved in the regulation of the expression of osteoblast bone-matrix structural-protein genes such as type 1 collagen or ALP. Based on the obtained results, it could be hypothesized that the high biological activity of the extracts obtained from fermented sprouts was connected with the presence of large amounts of coumestrol, formononetin and biochanin A. The pro-osteogenic potential of these isoflavonoids was associated with the activation of ALP, as well as the upregulation of *RUNX2* and *COL1A* mRNA levels in Saos-2 cells [29].

The chronic generation of reactive oxygen species by bone-modelling cells may accelerate the destruction of calcified tissue. Preventing intensified ROS generation could be one of the crucial mechanisms protecting bone tissue against structural collapse and loss of bone mass. Oxidative stress has been identified as the main inhibitor of osteoblastic differentiation and osteoclast activation [30]. All the studied samples demonstrably decreased intracellular ROS levels, revealing cytoprotective properties. There is growing evidence that reactive oxygen intermediates, which are generated in large quantities as a result of oxidative stress, act as a signal for the release of cytokines [31,32]. Among pro-inflammatory cytokines, the most relevant for bone health are tumor necrosis factor α and interleukin-6. These stimulate osteoclastogenesis, enhance bone resorption and inhibit osteoblast function; their elevation is observed in osteoporosis [33]. TNF-α secretion was diminished in the cells exposed to the ChUF and ChUFH extracts. Thus, it can be suspected that C. arietinum constituents have antiresorptive potential. Data showed that the main isoflavonoid constituents of C. arietinum (coumestrol, genistein and daidzein) were able to diminish TNFα secretion, osteoclast formation and resorption in RAW 264.7 cells [34]. More detailed studies with Jurkat E6.1 T cells revealed that the reduction of TNF-α and IL-6 at the mRNA and protein levels was associated with decreased expression of RANKL [35].

Bone and breast tissues rely on estrogen, which stimulates bone mineralization but, at the same time, may induce breast malignancy in excess [19]. MCF-7 human breast-cancer cells are known to express both isoforms of estrogen receptors (ERα and ERβ) [29], and were thus used in a proliferation experiment to evaluate the estrogenic potency of C. arietinum extracts. Taking into account that bone is one of the most common metastatic sites for breast cancer [20], the effect of the C. arietinum isoflavones on MCF-7 cell migration was assessed. The C. arietinum sprout preparations were able to decrease migration without cell detachment. From the detected decrease in MMP2/9 at the transcriptional level, it can be supposed that the constituents of fermented C. arietinum influenced the expression and activity of these matrix metalloproteases. There is evidence that coumestrol, the main isoflavonoid in fermented extracts, reduced the migration of KGN granulosa-like tumor cells without affecting cell proliferation [36]. Because reactive oxygen species, especially H_2_O_2_, are the key promoting intermediators of migration, the extracts’ influence on intracellular ROS generation was assessed. A previous study with docking simulation and isothermal titration calorimetry (ITC) modelling demonstrated that formononetin and biochanin A induced significant conformational changes in actin, which, in turn, may reduce the actin reorganization of the cytoskeleton and cell movement [37]. Isoflavonoids’ antioxidant properties protect cells against the direct oxidation of cellular lipids, proteins and DNA [1,2], but the observed cytoprotection may also result from the activation of enzymes involved in the removal of ROS. The obtained results are also relevant regarding the conformational changes in actin known to be caused by isoflavonoids; such changes may influence cell movement [26]. It is known that the main phenolic components of extracts, such as coumestrol, formononetin and biochanin A, are effective scavengers of ROS able to modulate intracellular glutathione levels, as well as the activity of superoxide dismutase or cyclooxygenase [8]. Recent studies showed that the binding of coumestrol with bovine serum albumin preserved its antioxidant properties [38]. Previous results demonstrated that isoflavones from *T. pratense*, mainly formononetin and biochanin A, decreased ROS generation and hindered radical spreading via cellular-membrane stiffening, which was accompanied by the downregulation of MCF-7 cell migration [26]. A decrease in membrane fluidity, suppressing metastatic potential, was observed in PC3 cells treated with genistein and daidzein [39]. Based on this evidence, one can suspect that *C. arietinum*’s phenolic compounds may inhibit cell migration, partially acting as oxidative-stress reducers, factors downregulating MMP expression or modulators of cellular-membrane dynamics.

It is known that the activation of the ERα receptor induces cell proliferation, which further promotes tumor development, especially in the breast tissue [40]. The activation of ERβ by ligands was found to inhibit the proliferation of MCF-7 cells [41]; it could be supposed that unfermented *C. arietinum* extracts’ components bind with the ERβ isoform, balancing the ERα response and, ultimately, influencing the proliferation rate. There are studies demonstrating that isoflavones bind the ERβ isoform with greater affinity than ERα [16]. The presented results obtained for the fermented extracts at low concentration demonstrate their ability to stimulate the proliferation of MCF-7 cells, but these samples had no influence on the expression of ERα and ERβ receptors. Additionally, all the preparations were able to decrease the MCF-7 cell-migration rate. Taking into account the observed osteogenic properties of the fermented extracts, it needs to be emphasized that *C. arietinum* isoflavones targeted osteoblast differentiation and matrix mineralization without the activation of Saos-2 cell proliferation. Furthermore, Saos-2 cells were more resistant to *C. arietinum*’s cytotoxic activity than MCF-7 cells (Figure 4). Microscopic observations confirmed that the elevation of the ChUFH extract concentration to the highest non-cytotoxic dose (75 µg/mL) effectively increased Saos-2 matrix mineralization (Figure 7B), while simultaneously reducing the metabolic activity of MCF-7 in a significant manner. Therefore, it can be assumed that *C. arietinum* fermented sprouts at elevated doses (e.g., the studied 75 µg/mL dose) can stimulate osteoblast matrix mineralization without the activation of breast-cancer proliferation. It was also confirmed that a 40 µg/mL dose of the fermented samples induced the apoptotic form of cellular death in MCF-7 cells without any toxic effect on Saos-2 cells. Taking into account the activation of caspase-9, it can be presumed that mitochondrial disruption was involved in the observed process, as the metabolic activity was decreased. MCF-7 cells are known to lack caspase-3 expression [42], so the activity of downstream caspase-7 was observed, which was, in turn, activated by caspase-9. Proapoptotic potential against MCF-7 cells was also shown for *C. arietinum* sprouts rich in biochanin A, formononetin, ononin and biochanin A-7-o-β-d-glucoside [42]. The disruption of mitochondrial membrane potential was observed, followed by the increased expression of caspase-7 and caspase-9. Among the identified compounds, formononetin has been demonstrated as able to impair proliferation via apoptosis induction in ER-positive MCF-7 and T-47D cells [43]. Thus, *C. arietinum* sprout isoflavonoids can be potentially used at higher concentrations as preventive agents in the human diet, to combat ER-positive breast-cancer development; however, this hypothesis requires further in vivo research.

It needs to be emphasized that the *C. arietinum* preparations were used at concentrations without cytotoxic impacts on cells (IC_0_ = 25 µg/mL) in the present study. Previous research showed that a daily intake of 5 mg of biochanin A (which corresponds to 1 µM) prevented bone loss [44]; thus, effective concentrations of the analyzed isoflavones seem to be achievable under physiological conditions. Herein, it is worth mentioning that an elevated dose of fermented sprout extract (75 µg/mL) decreased the viability of MCF-7 cells (by circa 50%) without any cytotoxic effects on Saos-2 cell viability and mineralization. Regardless of the positive effects of the *C. arietinum* sprouts observed in vitro, the in vivo effectiveness of isoflavonoids is regulated by their bioavailability, which depends on their form. It is known that isoflavone glycosides must be metabolized by the intestinal microflora to be transformed into bioavailable forms [45]. The efficacy of microbiological transformation depends, in turn, on the overall population and composition of the intestinal microbiota, especially in older subjects with poor intestinal colonization. To avoid this disadvantage, the sprouts were hydrolyzed with β-glucosidase, which released the aglycones of formononetin and biochanin A’s methylated derivatives. Methylated derivatives are approximately three-times-better absorbed than unmethylated forms and are distributed in the plasma [45]. Further metabolism to genistein and daidzein to equol in the next step can occur, but the rate of biotransformation depends on many factors; future studies on the dependence of the isoflavonoid extract composition, plasma concentration and action must therefore be performed in vivo with many subjects. The presented in vitro study based on animal cell cultures and extracts obtained from plant material is, therefore, the first step, which is necessary for performing further in vitro digestion experiments or considering clinical trials. Other components of the extracts, especially soluble proteins, carbohydrates and soluble dietary fiber, may also influence the bioavailability and metabolism of phenolic compounds after oral ingestion. Therefore, it is very difficult to compare the pharmacokinetics of a pure isoflavone compound, with even its identified metabolite and extract component being a composite matrix. A study performed on soy protein containing isoflavone glycosides suggested that the glycosidic group delays the degradation of isoflavones, leading to the higher bioavailability of their aglycones or equol [46].

Functional foods with health-promoting and disease-preventing effects are currently being studied. *C. arietinum* sprouts, especially after fermentation with *Lactobacillus casei*, could be considered as a dietary component of functional and little-processed food able to prevent the loss of bone mass induced by the chronic inflammation connected with obesity. Furthermore, a diet rich in phytoestrogens (especially soy isoflavones) has been documented as preventive for postmenopausal osteoporosis and breast cancer [17,47]. In this regard, it was shown that biochanin A, one of the components of *C. arietinum* sprouts, may decrease osteoclast activation, osteoporosis and mammary carcinogenesis via interaction with RANKL [29,44,48].

## 4. Materials and Methods

### 4.1. Chemicals and Reagents

All chemicals used, if not stated otherwise, were obtained from Sigma-Aldrich (St. Louis, MO, USA).

### 4.2. Chickpea Extracts and Isoflavonoid Determination

*Cicer arietinum* L. seeds were obtained from FN Granum (Wodzierady, Poland), and their germination and fermentation were carried out with *Lactobacillus casei* 0979 strain, as described previously [17]. UVB light was chosen for sprouting after a preliminary study [17]. Four preparations of isoflavones were obtained as follows: chickpeas sprouted by UVB radiation (ChU), chickpeas sprouted by UVB radiation and hydrolyzed with β-glucosidase (ChUH), fermented sprout phenolic extract hydrolyzed by β-glucosidase (ChUF) and fermented sprout phenolic extract hydrolyzed by β-glucosidase (ChUFH). Briefly, the sprouts were freeze dried (20 h, 0.340 mbar, and 4 h, 0.250 mbar) in a DELTA 1-24 LSC Christ (Osterode am Harz, Germany), and ground in a laboratory mill IKA type A 11 (Staufen, Germany), with a 2 mm screen mesh. The extraction was carried out in a pressure Speed Extractor E-916 Buchi (Essen, Germany). The extraction temperature was 50 °C, and the pressure, 10 MPa. A sample of 0.5 g of the milled lyophilized sprouts was placed in the extraction cell with a capacity of 60 mL. One cycle of extraction was performed with a mixture of solvents composed of methanol/water/acetic acid (90:9:1, *v*/*v*/*v*). The resulting extracts were quantitatively transferred to a volumetric flask, made up to a volume of 50 mL and filtered through a syringe cellulose filter with a pore diameter of 0.2 μm. Then, an aliquot of the sample was analyzed with an LC-ESI-MS system.

The LC-ESI-MS analysis of isoflavones was performed in accordance with Gao et al. [21] with some modifications and by using a Shimadzu (Kyoto, Japan) liquid chromatograph equipped with a SPD M20A diode array detector and a quadrupole mass spectrometer 2020, with an ion source of electrospray type (ESI), in positive-scan mode. A Kinetex C18 column (5 µm, 150 × 2.1 mm, Phenomenex, Torrance, CA, USA) was used. The column was thermostated at 30 °C. The sample was placed in the autosampler, and 10 µL was injected. Gradient elution was performed at a mobile-phase flow rate of 0.4 mL/min. Phase A was water/acetic acid at 98:2 (*v*/*v*), and phase B was acetonitrile/acetic acid at 98:2 (*v*/*v*). The gradients used were as follows: 0 min—10% B; 5 min—20% B; 10 min—45% B; 14 min—50% B; 16 min—100% B; 17 min—100% B; 18 min—10% B; 19 min—100% B. Identification and quantification were carried out using external calibration curves of the reference standards—daidzin ≥ 95%, daidzein ≥ 98%, ononin ≥ 99%, sissotrin ∼95%, genistin ≥ 98%, genistein ≥ 98%, biochanin A ≥ 95%, formononetin ≥ 99%, glycitin ≥ 98% and coumestrol ≥ 95%—which were purchased from Sigma Aldrich (St. Louis, MO, USA). The calibration ranges amounted 0.05–0.50 mg/mL. The following conditions were used for ESI-MS analysis: voltage at the interface, 4500 kV; interface temperature, 350 °C; temperature of the desolvation line, 250 °C; spired gas flow, 1.5 L/min; temperature of the heating block, 400 °C; drying gas flow, 15 L/min; operation, SIM mode (Selected Ion Monitoring); scavenging at *m*/*z* [m + 1] typical for isoflavones of red clover: 255 (daidzein), 269 (coumestrol), 270 (formononetin), 271 (genistein), 285 (biochanin A), 417 (daidzin), 431 (ononin), 433 (genistin) and 447 (sissotrin). The system used the LabSolutions control software (Shimadzu, Kyoto, Japan). The amount of identified isoflavones was calculated based on dry mass of sprouts or extracts and is presented in Table 1.

### 4.3. Cell Culture and Exposure Conditions

All the cell-culture reagents were obtained from Life Technologies (Carlsbad, CA, USA), while the tissue-culture plastics were supplied by Greiner Bio-One GmbH (Frickenhausen, Austria). All the experimental measurements were performed using the Synergy 2 BioTek microplate reader (BioTek, Winooski, VT, USA). Microscopic observations of cell migration were performed using an MDG41 Leica (Leica Microsystems, Wetzlar, Germany) under 10× magnification; cell mineralization and alkaline phosphatase (ALP) activity were observed with a Nikon TS100 Eclipse (Tokyo, Japan) microscope under 200× magnification.

The human breast cancer MCF-7 cell line was cultured in Dulbecco′s modified Eagle′s medium (DMEM), containing 10% fetal bovine serum (FBS), 100 U/mL penicillin, 100 μg/mL streptomycin and 25 μg/mL amphotericin B. Human osteosarcoma Saos-2 cells were cultured in DMEM Low Glucose with 10% FBS medium, supplemented with 100 U/mL penicillin, 100 μg/mL streptomycin and 25 μg/mL amphotericin B. After 24 h, the osteogenesis process was induced with medium consisting of DMEM Low Glucose supplemented with 2-phospho-l-ascorbic acid (100 μM), l-proline (34.8 μM) and β2-glycerol phosphate (5 mM) [49]. The medium was changed every two days and contained the test compounds. After 7 days, the measurements of mineralization were performed.

The cells were maintained at 37 °C in a humidified incubator containing 5% CO_2_. The test extracts were obtained by dissolving lyophilized sprout extracts in PBS/DMSO (1:1 *v*/*v*) at 300 mg/mL, resulting in 13.28–39.31 μmol/mL of isoflavones, which were further diluted with culture medium. The control cells were treated with a corresponding volume of medium, with the addition of 50% DMSO; the final concentration of DMSO in the cell-culture medium did not exceed 0.1%. The extracts’ concentrations used in the biological studies are presented in the descriptions of the tests carried out. Unless otherwise stated, cells were seeded into 96-well plates at 1 × 10^4^ cells per well for the experiments.

### 4.4. Cell Metabolic Activity and Proliferation

The extracts’ influence on the metabolic activity of cells was measured with Presto Blue reagent. In this assay, the intensity of the resorufin fluorescence produced from resazurin by mitochondrial enzymes correlates with the number of living cells. Cells were seeded in complete medium and grown for 24 h; then, the medium was changed for starvation medium (with 0.1% FBS), and the cells were incubated in the presence of the studied extracts diluted in culture medium for 48 h. Saos-2 cells were incubated with extracts in complete medium and grown for 24 h; the medium was then changed for osteogenic medium, and the cells were incubated in the presence of the studied extracts diluted in culture medium for 96 h. Cell metabolic activity was quantified according to the manufacturer’s instructions, by measuring the fluorescent signal at F530/590 nm.

To study the extracts’ influence on cell proliferation, the CyQuant Proliferation Assay (Life Technologies) was used, which allows the measurement of cellular DNA content based on the amount of fluorescent cyanine dye bound to nucleic acids. After the addition of the kit components for 1 h, the fluorescent signal at F485/528 nm was measured.

### 4.5. Alizarin Red Cell Staining

Since ALP is involved in matrix mineralization, the amount of calcium deposited in the Saos-2 cells’ extracellular matrix was quantified after cell staining with Alizarin Red S dye according to Muthusami et al. [50] with modifications. Briefly, after 7 days of differentiation and treatment with samples, cells were washed with PBS, fixed with 5% formaldehyde solution for 30 min at room temperature and then incubated for 30 min in 1% Alizarin Red S (2% ethanol, pH 4.0) solution. After rinsing with distilled water (2×), the cells were observed under a microscope. To quantify matrix mineralization, calcium-bound Alizarin Red S was solubilized with 100 mM cetylpyridinium chloride (100 µL) by plate shaking, and after 1 h, the absorbance at 570 nm was measured.

### 4.6. Estimation of ALP Activity

After the treatment, Saos-2 cells were rinsed with PBS; then, 1.0 mg/mL *p*-nitrophenyl phosphate (*p*-NPP), which is a substrate for alkaline phosphatase, in 0.2 M Tris buffer was added. After 15 min of incubation at 37 °C, the absorbance at 405 nm was measured. For the microscopic visualization of ALP activity, BCIP (5-bromo-4-chloro-3-indolyl phosphate)/NBT (nitroblue tetrazolium) substrate was added. In this assay, ALP activity is evaluated by the appearance of a cellular dark-blue product generated by the enzyme from the BCIP/NBT substrate. After 30 min of incubation, the cells were photographed.

### 4.7. Determination of Interleukin-6 and Tumor Necrosis Factor α

After Saos-2 cell treatment, the medium was collected and the protein concentrations of IL-6 (Human IL-6 ELISA kit, Biorbyt Ltd., Cambridge, UK) and TNF-α (Human TNF alpha ELISA kit, Biorbyt Ltd., Cambridge, UK) were determined using ELISA kits, following the manufacturer’s instructions. In cell lysates obtained with 0.1% Triton X-100 in PBS, the protein level was quantified using the Protein Assay Dye Reagent Concentrate (Bio-Rad Laboratories GmbH, München, Germany), and the absorbance values were normalized to protein content. The obtained values were used to calculate cytokine secretion, expressed as the percentage of the secretion of the untreated control cells (cells treated with an equal volume of the vehicle instead of the preparation).

### 4.8. Cell Migration Assay

The effects of the extracts on cellular migration was evaluated with a modified scratch-wound assay, which is based on measuring the free detection zone: the standardized round-shaped area decreases with cell migration [51]. Briefly, cells were seeded overnight on 96-well plates with “stopper” barriers that created a central cell-free detection zone for the cell-migration experiments. The medium was changed to create starvation conditions (with medium containing 0.1% FBS) for 24 h, and then, the stoppers were removed to allow cell migration into the standardized detection zone. Photos of the zones were taken at 0, 24 and 48 h intervals. The migration of stimulated cells vs. unstimulated cells was calculated according to the formula:

(1) $$migration\;\left[\%\right]\;=\;\frac{V_{t0}-V_{ti}}{Vk_{t0}-Vk_{ti}}\;\times \;100\%\;$$

where *V_t_*_0_ is the area of the scratch for the stimulated samples at time 0 (mm^2^), *V_ti_* is the scratch area for the stimulated samples at 24 or 48 h (mm^2^), *Vk_t_*_0_ is the scratch area for the control samples at time 0 (mm^2^), and *Vk_ti_* is the scratch area for the stimulated samples at 24 or 48 h (mm^2^). Scratch-area reduction was determined according to the formula:

(2) $$scratch\;area\;\left[\%\right]\;=\;\frac{V_{t0}-V_{ti}}{Vk_{t0}-Vk_{ti}}\;\times \;100\%$$

### 4.9. Adhesion Assay

To determine the effects of the extracts on cell adhesion, cells were fixed after incubation in 4% paraformaldehyde for 15 min, stained with 0.05% crystal violet solution for 30 min and randomly photographed. To quantify the numbers of attached cells, the crystal violet was dissolved with 70% ethanol and the absorbance at 570 nm was measured [27].

### 4.10. Detection of Intracellular Reactive Oxygen Species Generation

To determine the effects of the extracts on the intracellular generation of ROS after 48 h of treatment with the extracts, cells were incubated with 10 μM dichloro-dihydro-fluorescein diacetate (DCFH-DA) probe for 30 min. The fluorescent signals at λ_ex_ 485 nm and λ_em_ 530 nm were analyzed. The negative control contained only cells in culture medium (with vehicle), while the positive control contained 500 µM *tert*-butylhydroperoxide (*t*-BOOH) [52,53].

### 4.11. Detection of Apoptosis Induction

Apoptosis induction was studied in MCF-7 cells after 48 h of incubation with samples at 40 µg/mL. To quantify the amounts of phosphatidylserine (PS) released by apoptotic cells, the Annexin-V-FITC assay kit (Cayman Chemical, Ann Arbor, MI, USA) was used. After incubation, the cells were washed with phosphate buffer solution and stained for 10 min with 0.25 μg/mL Annexin-V-FITC (fluorescein isothiocyanate). The level of PS was measured by the change in fluorescent signal at λ_ex_ 485 nm and λ_em_ 530 nm. To detect membrane permeabilization, 1 μg/mL propidium iodide (PI) was used; after cell incubation for 30 min, the fluorescent signals at λ_ex_ 535 nm and λ_em_ 620 nm were measured.

To detect caspase activation, the Apo-ONE Homogeneous Caspase-3/7 Assay (Promega Corporation, Madison, WI, USA) was used. The fluorescence generated from the Z-DEVD-R110 substrate by caspases was measured at λ_ex_ 485 nm and λ_em_ 530 nm. The activation of caspase-9 was measured after 45 min of cell incubation with a luminogenic substrate containing the LEHD sequence, in the Caspase-Glo 9 Assay, according to the manufacturer’s instructions.

### 4.12. Measurement of Glutathione Peroxidase Activity and H_2_O_2_ Levels

The effects of the extracts on the activity of glutathione peroxidase (GPx) were studied with a Glutathione Peroxidase Assay kit, according to the manufacturer’s instructions. A Hydrogen Peroxide Colorimetric Assay (Sigma-Aldrich, Steinheim, Germany) was used to determine the level of hydrogen peroxide according to the manufacturer’s instructions. After cell treatment, the hydrogen peroxide color reagent was added, and the absorbance was measured at 550 nm.

### 4.13. Gene Expresssion Analysis

Cells were seeded into a 6-well plate at a density of 2 × 10^5^ cells/well. Total RNA was extracted from MCF-7 cells after 48 h of incubation with extracts in starvation medium and from Saos-2 cells after 7 days of treatment. A GeneMatrix Universal RNA Purification Kit (Eurex Ltd., Gdansk, Poland) was used for RNA isolation according to the manufacturer’s procedure. The RNA samples were purified with Amplification Grade DNase I and reverse transcribed with an NG dART RT Kit (Eurex Ltd., Gdansk, Poland). Real time RT-PCR was carried out using the SG qPCR Master Mix (Eurex Ltd., Gdansk, Poland) on a BioRad CFX96 qPCR System (Bio-Rad, Hercules, CA, USA). Complementary DNA representing 6 ng of total RNA per sample was subjected to 25–40 cycles of PCR amplification. The samples were first incubated at 95 °C for 40 s; then, at 55 °C for 30 s; and finally, at 72 °C for 30 s. To exclude non-specific products and primer dimers, after the cycling protocol, melting-curve analysis was performed by maintaining the temperature at 52 °C for 2 s, followed by a gradual temperature increase to 95 °C. The threshold cycle (Ct) values for that gene were comparable across experiments performed independently. The level of target-gene expression was calculated as 2^−ΔΔCt^, where ΔΔCt = [Ct(target) − Ct(reference gene)]_sample_ − [Ct(target) − Ct(reference gene)_control_]. Gene expression in MCF-7 cells was normalized using the constitutively expressed hypoxanthine phosphoribosyltransferase 1 (*HPRT1*) as a reference gene. The following primer sequences were used to determine the genes’ expression: *MMP2*, 5′-CTCATCGCAGATGCCTGGAA-3′ (F) and 5′-TTCAGGTAATAGGCACCCTTGAAGA-3′ (R); *MMP9*, 5′-ACGCACGACGTCTTCCAGTA-3′ (F) and 5′-CCACCTGGTTCAACTCACTCC-3′ (R); *HPRT1*, 5′-TGACCAGTCAACAGGGGACA (F) and 5′-AAGCTTGCGACCTTGACCAT-3′ (R). Gene expression in Saos-2 cells was normalized using the constitutively expressed glyceraldehyde-3-phosphate dehydrogenase (*GAPDH*) as a reference gene. The following primer sequences were used to determine the genes’ expression: *RUNX2*, 5′-CAGTTCCCAAGCATTTCATCC-3′ (F) and 5′-TCAATATGGTCGCCAAACAG-3′ (R); *ALP*, 5′-ACCTCGTTGACACCTGGAAG-3′ (F) and 5′-CCACCATCTCGGAGAGTGAC -3′ (R); *COL1A1*, 5′-GCCAAGACGAAGACATCCCA-3′ (F) and 5′-CACCATCATTTCCACGAGCA-3′ (R); *RANKL*, 5′-GAGTTGGCCGCAGACAAGA-3′ (F) and 5′-TTGGAGATCTTGGCCCAACC-3′ (R); *GAPDH*, 5′-CCACCCATGGCAAATTCCATGGCA-3′ (F) and 5′-TCTAGACGGCAGGTCAGGTCCACC-3′ (R). Data analyses were performed based on at least three independent experiments.

### 4.14. Western Blot Analysis

Cells were seeded into a 6-well plate at a density of 2 × 10^5^ cells/well. After cell incubation for 48 h with extracts, the cells were lysed with M-PER Mammalian Protein Extraction Reagent with Halt protease inhibitor (ThermoFisher Scientific, Waltham, MA, USA). Equal aliquots (20 μg) of the protein samples were separated by 4–12% SDS-PAGE, transferred to nitrocellulose membranes (BioRad, Hercules, CA, USA) and blocked with 5% BSA in TBST buffer. The membranes were incubated with rabbit β-actin (Cell Signaling) and ERα/ERβ (ThermoFisher Scientific) antibodies at 4 °C overnight, and then incubated with horseradish peroxidase-conjugated mouse anti-rabbit secondary antibody (Cell Signaling Technology, Danvers, MA, USA) for 1 h at room temperature. The Western blots were developed using SuperSignal™ West Pico Chemiluminescent Substrate (ThermoFisher Scientific) and quantified by densitometry.

### 4.15. Statistical Analysis

All data expressed as mean ±standard deviation (S.D.) were subjected to statistical analysis. The determination of average values and one-way ANOVA analysis followed by Dunnett’s test were performed using GraphPad Prism 6.0 (GraphPad Software, Inc., La Jolla, CA, USA). Results were considered statistically significant when *p* ≤ 0.05.

## 5. Conclusions

This is the first study to demonstrate the effects of fermented *C. arietinum* sprouts on osteosarcoma Saos-2 and breast cancer MCF-7 cells. The extracts obtained from fermented sprouts had large amounts of isoflavonoids, mainly coumestrol, formononetin and biochanin A.

Extracts obtained from fermented *C. arietinum* sprouts, rich in phytoestrogens, were found to stimulate Saos-2 cell mineralization, which was followed by an elevation of ALP activity and upregulation of *RUNX2* and *COL1A* expression at the mRNA level. The *C. arietinum* isoflavonoids simultaneously diminished the pro-inflammatory response via the reduction of IL-6 and TNF-α cytokine secretion in Saos-2 cells, which may limit the activation of bone resorption. The migration of MCF-7 cells was also simultaneously inhibited.

Despite its cytoprotective activity, *C. arietinum*, at low concentration, showed a stimulatory effect on the proliferation of estrogen-dependent MCF-7 cells, whereas higher doses induced apoptosis. These results are important for carefully considering the use of fermented *C. arietinum* sprouts as dietary supplements for the prevention of osteoporosis. Nevertheless, literature data analysis confirms that the observed in vitro biological potential of fermented sprouts was comparable to data obtained for soya. This makes *C. arietinum* fermented sprouts a novel and rich source of active dietary phytocompounds. Additionally, fermented sprouts are a potent source of probiotic bacteria, which are not only beneficial for human health, being inhabitants of the gastrointestinal tract, but also could minimize the risk of sprouts contamination with pathogens. Still, the impact on bone mineralization observed in vitro of fermented *C. arietinum* sprouts needs further study, with pure molecules to assert the presence of a possible synergistic effect and chemical interaction between the identified constituents. Future studies should also focus on the activity of the obtained preparations after their in vitro digestion or treatment with gut microflora.

## Figures and Tables

**Table 1:** Isoflavone composition of *C. arietinum* sprouts; n.d.—not detected; values are means ± standard deviations, *n* ≥ 9; values with the same superscript (^a^, ^b^) in the same column within sprouts or extracts are not significantly different at *p* ≤ 0.05.

mg of Isoflavonoids Per 100 g of Dry Mass of Sprouts, (mg/100 g of Dry Mass)												
	Daidzin	Ononin	Glycitin	Genistin	Sissotrin	Daidzein	Biochanin A	Genistein	Formononetin	Coumestrol	Glycitein	Total Isoflavonoids
Chickpea/UVB (ChU)	1.17 ± 0.24	43.99 ± 21.38	4.37 ± 0.64	0.80 ± 0.06 ^a^	38.27 ± 4.07	n.d.	72.79 ± 6.12	0.53 ± 0.07	216.18 ± 18.33	12.18 ± 2.25 ^a^	n.d.	490.28
Chickpea/UVB/H (ChUH)	n.d.	n.d.	n.d.	n.d.	n.d.	0.71 ± 0.04	97.16 ± 8.71	1.03 ± 0.09	305.93 ± 25.15	12.18 ± 3.60 ^a^	2.78 ± 0.34	419.79
Chickpea/UVB/F (ChUF)	0.04 ± 0.01	08.85 ± 15.92	0.57 ± 0.07	0.76 ± 0.09 ^a^	22.16 ± 3.13	n.d.	256.05 ± 18.19 ^a^	12.18 ± 1.05 ^a^	676.41 ± 58.28	1017.92 ± 92.26 ^b^	n.d.	2094.92
Chickpea/UVB/F/H (ChUFH)	n.d.	n.d.	n.d.	n.d.	n.d.	0.02 ± 0.01	270.16 ± 21.26 ^a^	12.66 ± 0.94 ^a^	744.25 ± 60.27	1017.92 ± 88.82 ^b^	0.36 ± 0.05	2045.36
**mg of Isoflavonoids Per 100 g of DM (Dry Mass of Extracts)**												
hU	3.70 ± 0.52	54.94 ± 32.18	13.81 ± 1.10	2.53 ± 0.38	120.92 ± 10.93	n.d.	229.98 ± 27.39	1.67 ± 0.24	683.03 ± 53.30	38.48 ± 2.75 ^a^	n.d.	1549.07
ChUH	n.d.	n.d.	n.d.	n.d.	n.d.	2.08 ± 0.18	284.51 ± 29.04	3.02 ± 0.38	895.84 ± 74.49	35.67 ± 2.87 ^a^	8.14 ± 0.67	1229.25
ChUF	0.07 ± 0.02	88.29 ± 15.83	0.99 ± 0.07	1.31 ± 0.17	38.33 ± 5.91	n.d.	442.92 ± 35.91 ^a^	21.07 ± 3.07 ^a^	1170.06 ± 87.29 ^a^	1760.80 ± 130.54 ^b^	n.d.	3623.80
ChUFH	n.d.	n.d.	n.d.	n.d.	n.d.	0.03 ± 0.01	456.20 ± 40.28 ^a^	21.38 ± 3.07 ^a^	1256.75 ± 114.80 ^a^	1718.88 ± 103.35 ^b^	0.61 ± 0.04	3453.83
